# Oriented Antibody Coupling to an Antifouling Polymer Using Glycan Remodeling for Biosensing by Particle Motion

**DOI:** 10.1021/acs.bioconjchem.4c00196

**Published:** 2024-07-01

**Authors:** Maud D. M. E. Linssen, Yu-Ting Lin, Sebastian A. H. van den Wildenberg, Marrit M. E. Tholen, Arthur M. de Jong, Menno W. J. Prins

**Affiliations:** † Department of Biomedical Engineering, Eindhoven University of Technology, Eindhoven 5612AE, The Netherlands; ‡ Institute for Complex Molecular Systems (ICMS), Eindhoven University of Technology, Eindhoven 5612AE, The Netherlands; § Department of Applied Physics, Eindhoven University of Technology, Eindhoven 5612AE, The Netherlands; ∥ Helia Biomonitoring, Eindhoven 5612AR, The Netherlands

## Abstract

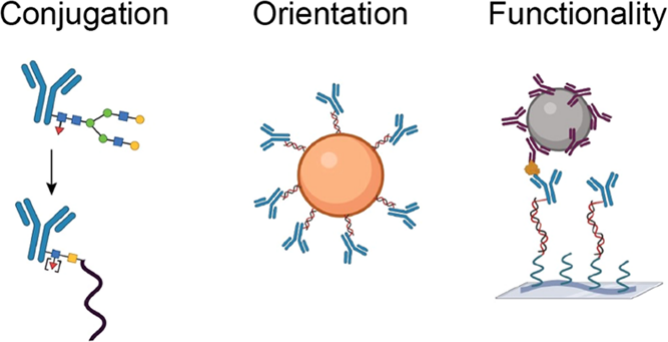

Biosensors based on immobilized antibodies require molecular strategies that (i) couple the antibodies in a stable fashion while maintaining the conformation and functionality, (ii) give outward orientation of the paratope regions of the antibodies for good accessibility to analyte molecules in the biofluid, and (iii) surround the antibodies by antibiofouling molecules. Here, we demonstrate a method to achieve oriented coupling of antibodies to an antifouling poly(l-lysine)-*grafted*-poly(ethylene glycol) (PLL-*g*-PEG) substrate, using glycan remodeling to create antibody–DNA conjugates. The coupling, orientation, and functionality of the antibodies were studied using two analysis methods with single-molecule resolution, namely single-molecule localization microscopy and continuous biosensing by particle motion. The biosensing functionality of the glycan-remodeled antibodies was demonstrated in a sandwich immunosensor for procalcitonin. The results show that glycan-remodeled antibodies enable oriented immobilization and biosensing functionality with low nonspecific binding on antifouling polymer substrates.

The growing interest in the continuous monitoring of molecular markers in healthcare applications inspires the development of biosensors that can measure biomolecular substances in a continuous fashion. Sensors for continuous monitoring are already widely available for glucose,^1^ a metabolite that is present at millimolar concentrations and can be measured by enzyme-based electrochemical sensing methods. However, other analytes such as hormones, proteins, and oligonucleotides are present at concentrations orders of magnitude lower, which cannot be measured by such methods. Analytes at micromolar concentrations and below can be detected by exploiting biomolecular binding rather than enzymatic conversion, using molecular binders such as antibodies (Abs) and aptamers. Continuous sensing using such affinity binders has been reported, e.g., using electrochemical aptamer-based techniques,^2−4^ fluorescence detection,^5,6^ and biosensing by particle motion (BPM).^7,8^

Antibodies are very relevant affinity binding molecules for biomolecular sensors because they are available for a wide variety of analytes and show good specificity and stability in biological fluids.^9−12^ In a biosensor, the antibodies are coupled to a sensing element such as a substrate or to particles. A commonly applied method to couple antibodies to a solid carrier is physical adsorption, based on electrostatic charge, hydrophobic interactions, van der Waals interactions, and hydrogen bonds.^13^ Physical adsorption is easy and quick, but the immobilized antibodies are randomly oriented, the binding functionality of the antibodies can be affected, and the coupling between the antibody and solid substrate can be rather weak, leading to a loss of antibodies over time.^14,15^ Stronger coupling while maintaining antibody functionality can be achieved by covalent coupling methods, such as using cross-linkers that target amines, carboxylic acid, or thiols, using Fc binding proteins or using oxidation of glycans,^9−12,16^ some of which enable directional coupling of antibodies^17−19^ and have been studied for improving biosensor sensitivity.^20−22^ Furthermore, an additional requirement for continuous sensing is that the sensor should give accurate results over long time spans in biological fluids, which implies that sensor functionalization strategies need to include antifouling properties. In summary, molecular architectures are needed that (i) couple antibodies in a stable fashion while maintaining the conformation and functionality, (ii) give outward orientation of the paratope region of the antibodies for good accessibility to analyte molecules in the biofluid, and (iii) surround the antibodies by antifouling molecules.

Here, we develop a strategy to achieve oriented antibody coupling on an antifouling polymer for continuous biosensing purposes. Oriented coupling is achieved by creating antibody–ssDNA conjugates via remodeling of the antibody glycans, followed by hybridization to ssDNA coupled to an antifouling poly(l-lysine)-*grafted*-poly(ethylene glycol) (PLL-*g*-PEG) coating on a solid substrate. The coupling strategy is demonstrated using anti-procalcitonin antibodies in a continuous biosensing technology with single-molecule resolution, called biosensing by particle motion (BPM).^8,23−25^ Procalcitonin (PCT) is an inflammation biomarker used to differentiate if a patient has a bacterial or viral infection and to monitor how a patient responds to antibiotic treatment.^26^ This paper studies the antibody modification method, investigates the orientation of the antibodies, demonstrates the implementation of the antibodies in a BPM biosensor, compares the proposed oriented antibody coupling with a nonoriented coupling method, and discusses the perspective of the substrate functionalization strategy.

## Results and Discussion

### Methodology

**Figure 1:**
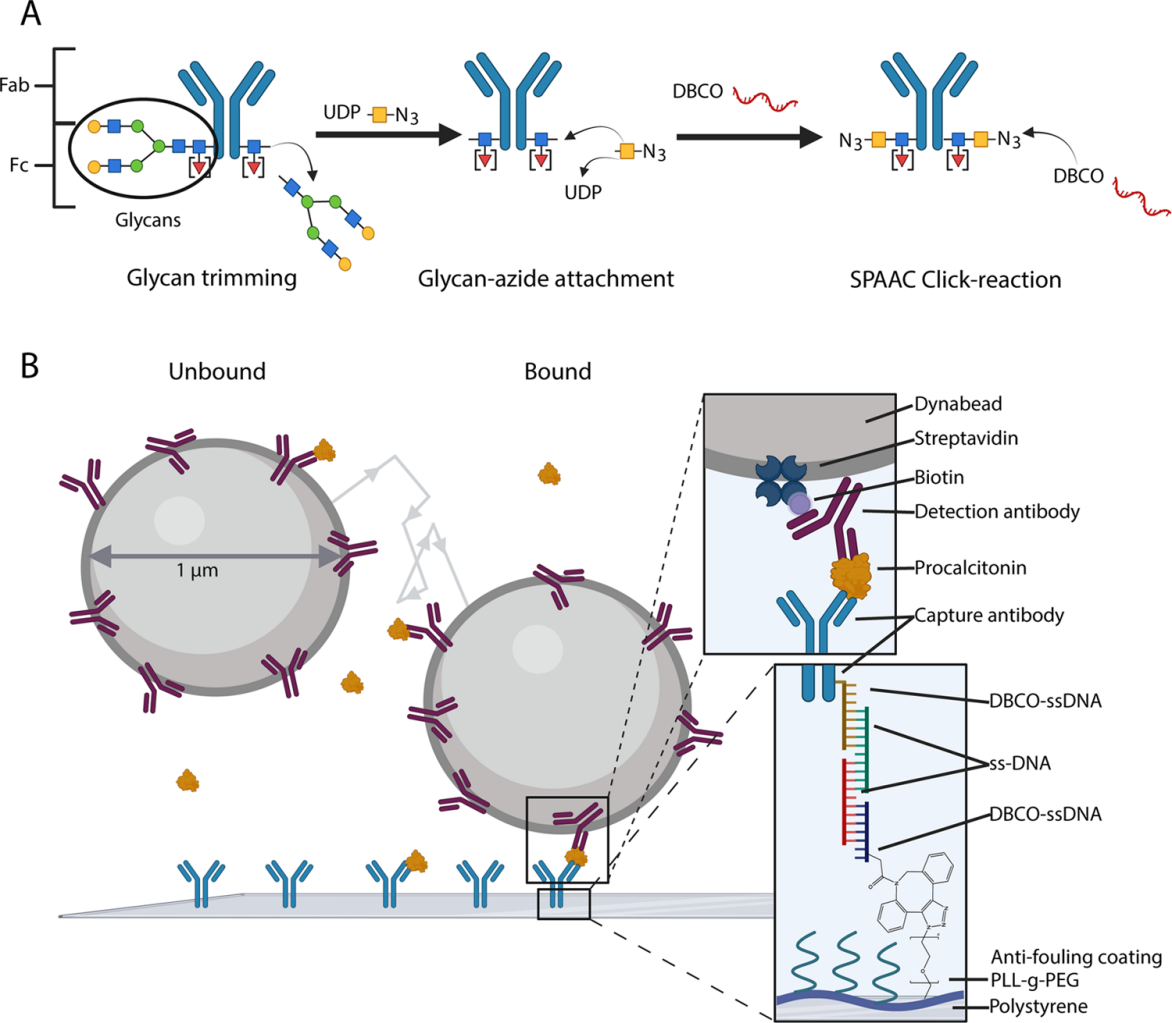
Biofunctionalization method based on glycan remodeling for oriented coupling of antibodies to an antifouling polymer in a continuous biosensor. (A) Antibody modification strategy. Antibodies contain glycosylation sites in the Fc region. Glycan remodeling is achieved by glycan trimming, using endoglycosidase SH, which cleaves the glycosidic bond between two *N*-acetylglucosamines (blue square). Thereafter, UDP-GalNac6N_3_ (UDP-yellow square-N_3_) is attached to the remaining *N*-acetylglucosamine by galactosyltransferase (Y289L) with loss of UDP. Finally, DBCO-ssDNA with a DBCO group at the 3′ end is attached to the azide via SPAAC click chemistry. (B) Implementation strategy of Ab–DNA conjugates in a biosensing by particle motion (BPM) sensor. The sensor uses the antibody-functionalized particles and substrate. The coupling strategy is shown in the blowups. The particles are functionalized with streptavidin, to which biotinylated antibodies are coupled. The substrate is functionalized with poly(l-lysine)-*grafted*-poly(ethylene glycol) (PLL-*g*-PEG) and PLL-*g*-PEG/N_3_. DBCO-ssDNA is coupled to the azide on the substrate via SPAAC click chemistry. The modified antibody is coupled to the DNA on the substrate via DNA hybridization, using two intermediate ssDNA strands. The DNA hybridization lengths are 25, 20, and 25 base pairs, from the substrate to the antibody, respectively. Analyte detection is based on tracking the motion of hundreds of particles simultaneously using dark-field video microscopy. Particles show free Brownian motion when unbound (panel (B) left). Upon formation of a sandwich bond (panel (B) right), the particles are bound and show confined Brownian motion. Created with BioRender.com.

Antibodies (Abs) are large proteins with a molecular weight of about 150 kDa, consisting of two identical heavy chains (HC, ±50 kDa) and two identical light chains (LC, ±25 kDa), linked via disulfide bridges. The antibody structure can be divided into a crystallizable fragment (Fc) and an antibody-binding fragment (Fab)^9−12,27^ (Figure 1A). All Abs are glycosylated at the Asn residue near amino acid 300, in the Fc domain,^27^ and about 20% of antibody types contain an additional glycosylation site in the Fab domain.^28^

The aim of this research is to couple antibodies in an oriented manner by modifying the glycans and by connecting the molecules via a DNA linker to an antifouling polymer. Ab–DNA conjugates were created using GlycoConnect (Figure 1A) that leaves the Fab site intact.^29,30^ First, the glycans were trimmed by endoglycosidase SH, which cleaves the glycans between the two *N*-acetylglucosamines. Then, UDP-GalNac6N_3_ was attached to the remaining *N*-acetylglucosamine by galactosyltransferase (Y289L) with loss of UDP. Finally, the conjugation of the DNA to the antibody was achieved by SPAAC click chemistry of DBCO-ssDNA to the azide group in the sugar.^31^ The antibodies were immobilized on a substrate functionalized with the antifouling polymer poly-l-lysine-*grafted*-polyethylene glycol/azide (PLL-*g*-PEG/N_3_).^32,33^ The immobilization was achieved by SPAAC click chemistry of DBCO-ssDNA to the azides on the substrate, followed by DNA hybridization of the Ab–DNA to the DNA on the substrate, using two intermediate ssDNA strands (Figure 1B).

The substrate functionalized with antibodies was used in a BPM sensor for measuring PCT in a sandwich format.^8^ BPM is based on tracking the mobility of micrometer-sized particles over time. Hundreds of particles are tracked simultaneously using dark-field video microscopy at 10× magnification. Images are obtained at 60 Hz for 5 min per measurement. The particles and substrate are functionalized with anti-PCT Abs. In the absence of an analyte, the particles exhibit free Brownian motion. When analytes are added, sandwich bonds are formed between the substrate-coupled Abs, PCT molecules, and particle-coupled Abs. A single sandwich bond restricts the motion of a particle; thus, confined Brownian motion is observed. The characteristic motion types—free or confined Brownian motion—are determined over time by quantifying the mean squared displacement (MSD) as a measure of the diffusivity of the particles. Particles with a high diffusivity (>0.15 μm^2^/s) exhibit free Brownian motion and are classified as unbound. Particles with low diffusivity (<0.15 μm^2^/s) show confined Brownian motion and are classified as bound. The diffusivity of hundreds of particles is assessed over time, and the temporal fraction of particles with a low diffusivity is reported as the bound fraction.^8^

In this publication, average values determined with an established technology are reported with the standard error of the mean (SEM, ) for an estimation of the true value. Average values are reported with the standard deviation (SD, ) if the width of the distribution is of importance.

### Antibody Modification

**Figure 2:**
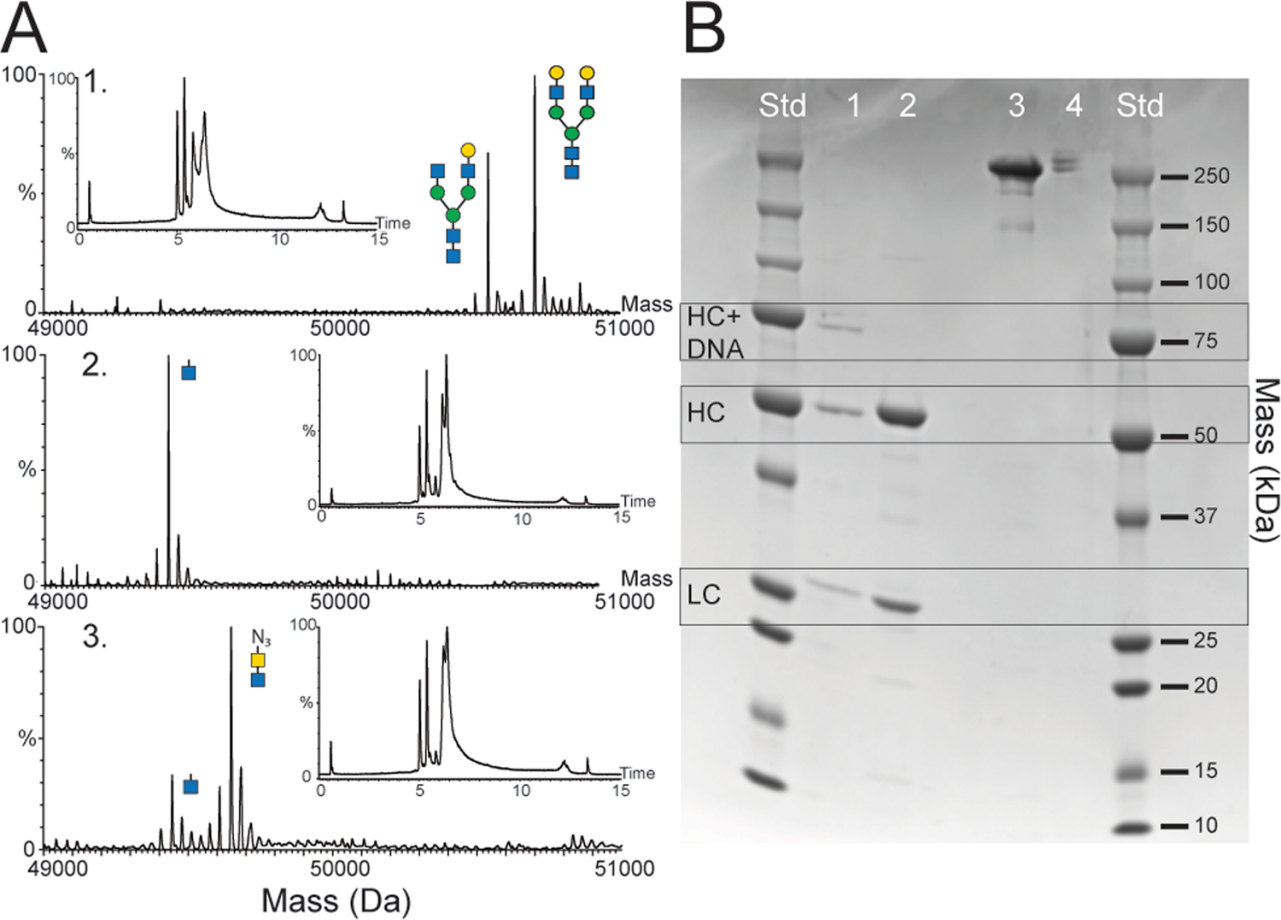
Glycan remodeling of anti-procalcitonin Ab in different stages. (A) Mass spectra of the heavy chain (HC) fragments of antibodies in different stages of glycan remodeling. The first spectrum shows intact glycosylation sites, the second spectrum shows antibodies after incubation with endoglycosidase SH, and the third spectrum shows antibodies after incubation with UDPGalNac6N_3_ and galactosyltransferase (Y289L), with a final glycan removal yield (±SEM) of 80.6 ± 0.5%. The mass spectra correspond to the masses detected in the chromatogram between 6 and 8 min. The chromatograms are shown in the insets of the corresponding mass spectra. (B) Sodium dodecyl sulfate polyacrylamide gel electrophoresis of (1) reduced remodeled Abs, with a HC-DNA conjugate at ±73 kDa, HC at ±50 kDa, and light chain (LC) at ±26 kDa; (2) reduced Abs, with HC at ±50 kDa and LC at ±25 kDa; (3) intact antibody; and (4) intact remodeled antibody. Antibody–DNA conjugation yield (±SD) is determined at 33 ± 2% and a ratio of DNA strands per antibody (2:1:0) at 10:45:45%.

The modification strategy was first tested and optimized using Trastuzumab. Subsequently, the modification was applied to anti-PCT Ab (13B9, HyTest). Trastuzumab was used for the initial study due to its availability in large amounts and because its Fc fragment is similar to the Fc fragment of the anti-PCT antibodies. The yield of the Ab modification was assessed using liquid chromatography/mass spectrometry (LC-MS) and sodium dodecyl sulfate polyacrylamide gel electrophoresis (SDS-PAGE). The results of the modification of Trastuzumab and optimization of the protocol are described in Supporting Information (SI) S2. For the remodeling of Trastuzumab, a glycan remodeling yield of 81% and an Ab–DNA conjugation yield of 65% were reached when using 4 mol equiv of DNA to Abs. The results of the anti-PCT Abs modification are shown in Figure 2.

Modification yield was assessed by analyzing the HCs of reduced Abs in different stages of the modification by LC-MS and SDS-PAGE and by analyzing the intact Abs by SDS-PAGE. The mass spectrum of reduced Abs shows the presence of HCs with a varying glycan pattern between 50.5 and 51 kDa (Figure 2A1), corresponding to the theoretical mass of the HC. After glycan removal, the reduced Abs (Figure 2A2) show a signal at 49.4 kDa and no signal between 50.5 and 51 kDa, indicating successful removal of all glycans. After coupling of the azide-containing sugar to the antibody, an additional signal at 49.6 kDa is observed (Figure 2A3). The increase in mass corresponds to the mass of the attached GalNac6N3 (203 Da). The signal at 49.4 kDa decreased in comparison to the spectrum of reduced antibodies (Figure 2A2) but did not disappear. The presence of this signal indicates that not all glycosylation sites are functionalized with azide-containing sugar. A modification yield (±SEM) of 80.6 ± 0.5% was determined by comparing the signal intensities of the mass spectra at 49.4 and 49.6 kDa. The complete removal of native glycans and over 80% attachment of azide-containing sugar to the glycans are in line with the findings of Wijdeven et al.^30^

In the SDS-PAGE gel (Figure 2B), the HC of reduced Abs is visible at ±50 kDa (lane 2, Figure 2B). After conjugation, the reduced Abs show bands at ±73 and ±50 kDa (lane 1, Figure 2B). The band at ±73 kDa indicates the formation of Ab–DNA conjugates. By analyzing multiple gels, a conjugation yield (±SEM) of 33 ± 2% was determined, without considering the modification yield discussed in the previous section. Nonreduced Abs and Ab–DNA conjugates were also analyzed in the gel (lane 3 and 4 respectively, Figure 2B). The nonreduced Abs show one broad band at the top of the gel, while for the Ab–DNA conjugate, three small bands are present at the top of the gel. The three bands indicate that about 10% of conjugates contain two DNA strands, 45% of conjugates contain one DNA strand, and 45% of the Abs are not conjugated to DNA. These results are in line with the conjugation yield of 33% of all HCs, as all Abs contain two HCs. The findings show that over 50% of the antibodies contain at least one DNA strand and are therefore suited for coupling to the sensor substrate. The conjugation yield for Ab–DNA conjugates is lower than for Ab–drug conjugates studied by Wijdeven et al.,^30^ who reported conjugation efficiencies larger than 66%. This difference may be caused by the larger size of the DNA in comparison to the small-molecule drug used by Wijdeven et al.^30^ Another difference is that surfactants were used by Wijdeven but not in our study. We note that the presence of a nonzero fraction of Abs without DNA are acceptable for a substrate biofunctionalization strategy, as unbound Abs are washed away after substrate functionalization.

### Orientation of Coupled Antibodies

**Figure 3:**
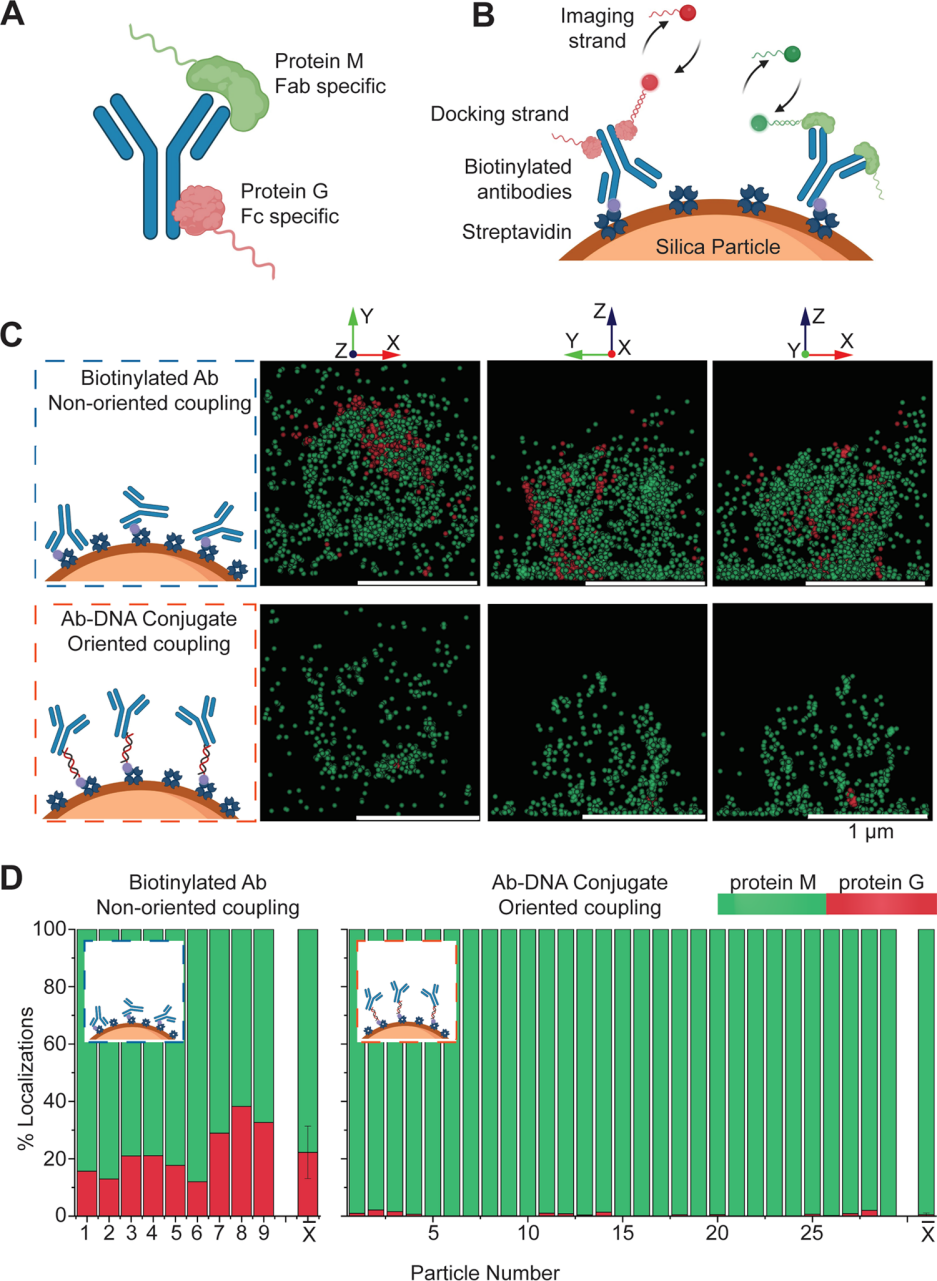
Single-molecule DNA-PAINT study on the orientation of antibodies coupled to streptavidin-functionalized silica particles, for nonoriented coupling using biotinylated antibodies and for oriented coupling using DNA-conjugated glycan-remodeled antibodies. Silica particles were used as carriers in this experiment because these have good imaging properties for DNA-PAINT. (A) ssDNA-conjugated protein M (green) and ssDNA-conjugated protein G (red) were used to localize Fab and Fc regions on the antibodies, respectively. (B) Schematic representation of two-color DNA-PAINT localization of protein M and protein G. ssDNA docking strands on protein M were imaged using imager strands with the green Cy3B fluorescent dye. ssDNA docking strands on protein G were imaged using imager strands with the red ATTO-647N fluorescent dye. (C) Left: schematic of streptavidin-coated silica particles functionalized with biotinylated Abs (left top) and DNA-hybridized Abs conjugates (left bottom). Right: for each functionalization, three-dimensional (3D) DNA-PAINT images of one particle are shown, projected on the *XY*, *YZ*, and *XZ* planes. All particles are shown in the SI (Figure S4). (D) Fraction of localizations in the green channel (protein M, Fab) and red channel (protein G, Fc). Left: particles with nonoriented coupling (*N* = 9). Right: particles with oriented coupling (*N* = 29). The last bar (*X̅*) represents the average % of localizations. Schematics created with BioRender.com.

The orientation of antibodies coupled to a solid carrier was studied using a single-molecule image method called DNA point accumulation for imaging in nanoscale topography (DNA-PAINT). The orientation of antibodies on particles was quantified using protein G and protein M, according to a method that was recently published by Tholen et al.^34^ We applied the imaging methodology to antibodies coupled to streptavidin-functionalized silica particles, as these have good imaging properties in DNA-PAINT. The particles were immobilized on a substrate, and accessible Fab and Fc domains of the antibodies on the particles were imaged using two-color DNA-PAINT. The imaging was achieved by targeting the Fc domains with protein G-ssDNA conjugates and the Fab domains with protein M-ssDNA conjugates (Figure 3A). The conjugated ssDNA strands function as docking strands for imaging by DNA-PAINT. The imaging technique uses the transient binding and unbinding of dye-labeled complementary imager ssDNA strands to localize the positions of the docking strands with nanoscale resolution.^35^ The ssDNA imager strands were functionalized with Cy3B (green) and ATTO-647N (red) dyes, in order to localize the docking strands coupled to the protein M and protein G, respectively, for quantification of the ratio of exposed Fab and Fc domains on the substrate of the particles (Figure 3B).

Two antibody coupling methods were studied: nonoriented coupling using Abs that were biotinylated by 1-ethyl-3-(3-(dimethylamino)propyl)carbodiimide-*N*-hydroxysuccinimide (EDC-NHS) chemistry and oriented coupling using Ab–DNA conjugates that were hybridized to a complementary biotin-DNA strand, as sketched in Figure 3C. The panels show projections of the 3D image of a single particle for both functionalization methods, projected onto three planes (*XY*, *YZ*, and *XZ*). 3D images were obtained using Bruker’s biplane technology. The orientation of the antibodies was determined from the number of green and red localizations. For Abs with EDC-NHS biotinylation (Figure 3C top), most of the localizations are in the green channel, indicating that predominantly, the Fab domains of the antibodies are accessible. However, some antibodies expose their Fc domains rather than the Fab domains, indicated by the red localizations. For Ab–DNA conjugates (Figure 3C bottom), nearly all localizations are green and hardly any red localizations are seen, indicating that Fab domains are accessible and hardly any Fc domains.

DNA-PAINT images of all studied particles are shown in the Supporting Information (Figure S4). The particles were analyzed for their fractions of green and red localizations (Figure 3D). On average (±SD), 77.8 ± 9.1 and 99.5 ± 0.6% of the localizations were green for particles functionalized with biotinylated Abs and Ab–DNA conjugates, respectively. This shows that for both coupling methods, more Fab domains are accessible than Fc domains. When a coupling method would give a random Ab orientation, 2:1 Fab-to-Fc ratio would be observed since every antibody has two Fab domains and one Fc domain, i.e., 67% of the localizations would be green. The biotinylated Abs give a higher fraction of green localizations in the DNA-PAINT experiments, indicating that the Abs have an outward orientation that is somewhat more favorable than random. In contrast, the fraction of green localizations is nearly 100% for the Ab–DNA conjugates, which demonstrates a very high orientation effectiveness of the Abs coupled via site-specific glycan remodeling.

### Antibody Functionality in a Biosensor

**Figure 4:**
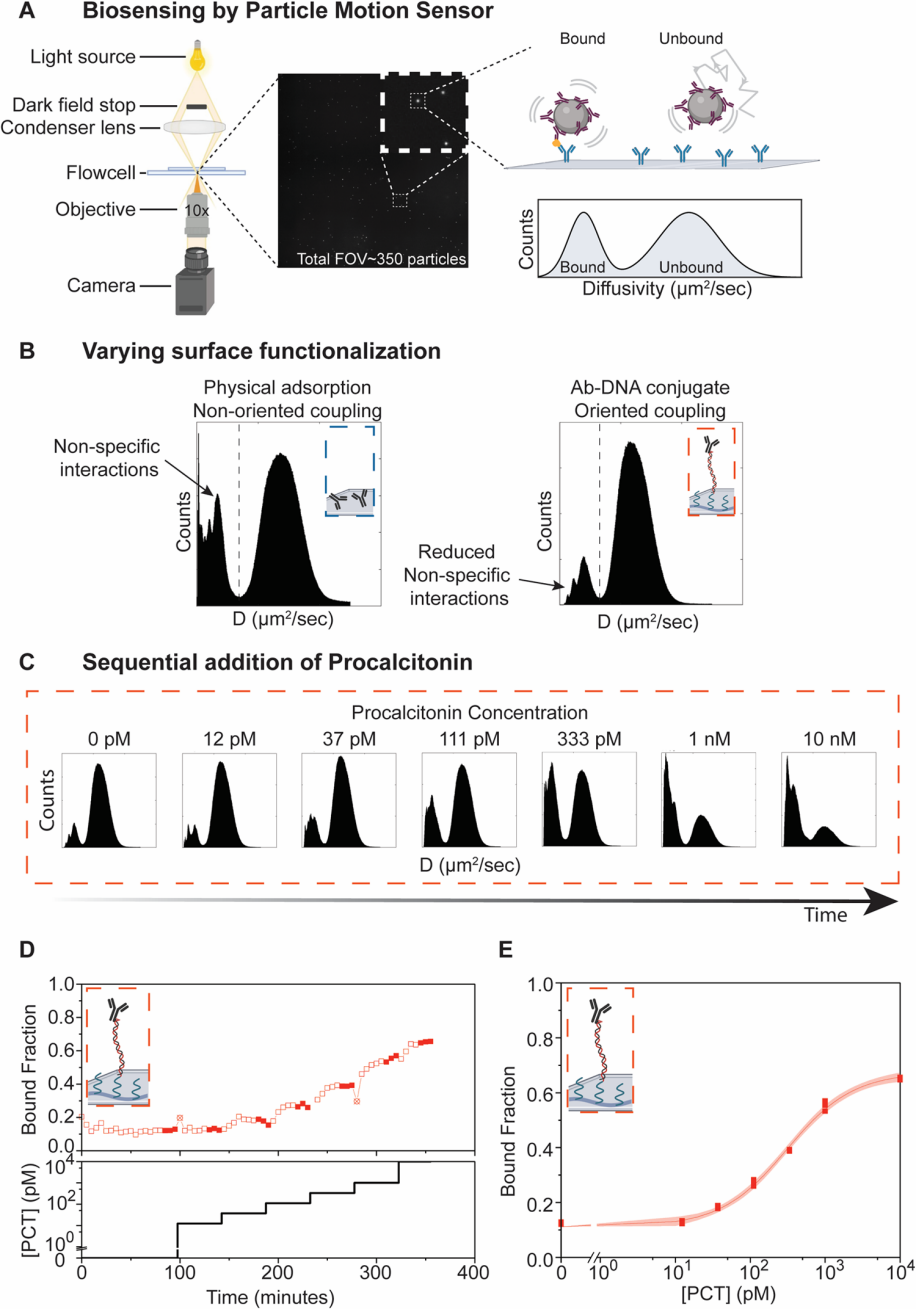
Antibody functionality studied in a biosensor based on particle motion (BPM). (A) In a BPM sensor, the movement of hundreds of particles is tracked using dark-field microscopy. The diffusive motion of all particles is plotted in diffusivity histograms. Particles with a low diffusivity (*D* < 0.15 μm^2^/s) are characterized as bound, and particles with a high diffusivity (*D* > 0.15 μm^2^/s) are characterized as unbound. (B) Diffusivity histograms for hundreds of particles in a BPM sensor with physically adsorbed antibodies on glass (left, *N* = 1020, BF = 0.26) and oriented coupled antibodies on PLL-*g*-PEG on polystyrene (right, *N* = 582, BF = 0.12). No analyte in solution. (C) Diffusivity histograms for a series of increasing analyte concentrations (procalcitonin) for a BPM sensor with orientally coupled glycan-remodeled antibodies on the substrate and biotinylated antibodies on the particles. (D) Data from the same experiment as in (C). Bottom panel: analyte concentration that was added to the BPM sensor. Top panel: measured bound fraction as a function of time. The filled red squares indicate the measurements that were included in (E). (E) Bound fraction as a function of analyte concentration, for the same experiment as in (C), using the filled red squares from (D). Data are fitted using , with a 95% confidence interval. *a* = 0.11 ± 0.01; *b* = 0.67 ± 0.01; *x*_c_ = 307 ± 22 pM; and *n* = 0.98 ± 0.07.

The biosensing functionality of the Ab–DNA conjugates coupled to the antifouling PLL-*g*-PEG coating was studied using procalcitonin as a model analyte and biosensing by particle motion as the readout method. In the experiment, the motion of hundreds of particles was tracked using dark-field microscopy, and the particles were characterized by their diffusivity^8^ (Figure 4A). Particles with a low diffusivity (*D* < 0.15 μm^2^/s) were characterized as bound, while particles with a high diffusivity (*D* > 0.15 μm^2^/s) were characterized as unbound. A sandwich sensor format was used, with particles functionalized using biotinylated Abs and the substrate using Ab–DNA conjugates, as illustrated in Figure 1B.

First, the degree of nonspecific binding was assessed by comparing two different substrate functionalizations: oriented Ab–DNA conjugates hybridized to the PLL-*g*-PEG coating on polystyrene and nonoriented physical adsorption of Abs on glass (Figure 4B). The bound fractions measured with a BPM sensor with Ab–DNA conjugates on PLL-*g*-PEG and physically adsorbed Abs were 0.12 and 0.26, respectively. For the sensor with Ab–DNA conjugates, the diffusivity histogram of the bound states shows a pattern of three peaks, with increasing height for increasing diffusivity, which indicates that some particles have single molecular bonds and other particles have multiple molecular bonds. The sensor with physically adsorbed Abs shows a broad distribution of diffusivity for the bound particles, with a distinct high peak at zero diffusivity, indicating that many particles show no movement at all. The substrate with Ab–DNA conjugates hybridized to the PLL-*g*-PEG shows less multivalent interactions and less nonspecific interactions than the physically adsorbed Abs. The lower amount of nonspecific interactions is likely due to the use of the antifouling PLL-*g*-PEG coating.^33^

The biosensing performance of the substrate functionalized with Ab–DNA conjugates hybridized to the PLL-*g*-PEG coating was tested by measuring a series of increasing PCT concentrations (Figure 4C–E). Control experiments without analyte showed a stable bound fraction (±SD) of 0.13 ± 0.02 over a period of 1.5 h. Subsequently, the PCT concentration was stepwise increased, with the sensor showing a response at PCT concentrations above 37 pM. A maximum bound fraction of approximately 0.65 was found at a PCT concentration of 10 nM.

The particle diffusivity histograms give insights into the binding behavior of the particles. With increasing PCT concentrations, single bonds form first, followed by multivalent binding of particles, with multivalent interactions dominating at and above 1 nM PCT (Figure 4C). Similar curves were obtained for physically absorbed Abs on the substrate; however, broad diffusivity distributions were observed for the bound particles due to nonspecific and multivalent bonds (Figure S5).

The increase in the signal in the BPM sensor with increasing PCT concentrations follows a sigmoidal curve (Figure 4E). The response was obtained using data at a stable sensor signal to exclude time effects (solid blocks of Figure 4D) and fitted using a sigmoidal fit with a 95% confidence interval following eq 1
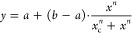
1In this equation, *y* is the bound fraction signal, *a* is the background signal of the curve, *b* is the signal value reached at the end of the curve, *x* is the analyte concentration, *x*_c_ is the analyte concentration at which half of the signal change is achieved [*y*(*x* = *x*_c_) = (*a* + *b*)/2], and *n* is the slope factor. The blank signal (±SD) is 0.11 ± 0.01, and the final bound fraction (±SD) at 10 nM is 0.67 ± 0.01. The curve shows a dynamic response in the high-pM and low-nM range. The dynamic response of the sensor can be adjusted by changing binder concentrations on the particles and on the substrate, as shown by Buskermolen et al.^36^

## Conclusions

We described a coupling methodology to achieve oriented coupling of antibodies to an antifouling substrate and demonstrated its biosensing functionality using a procalcitonin sandwich immunosensor. The oriented coupling was achieved by remodeling antibody glycans to contain an azide, attaching ssDNA by SPAAC click chemistry, and coupling the Ab–DNA conjugates to a PLL-*g*-PEG substrate via DNA hybridization. The glycan remodeling and conjugation of DNA to the antibodies were verified using SDS-PAGE and LC-MS, with a final conjugation yield of approximately 55%. The oriented coupling of the Ab–DNA conjugates was assessed in a DNA-PAINT experiment using Fc-binding protein G and Fab-binding protein M, with nonoriented Ab–biotin conjugates used as control. The DNA-PAINT results confirmed that the Ab–DNA conjugates are immobilized with an outward orientation of the paratope regions of the antibodies.

The functionality of the oriented antibodies was demonstrated in a biosensor based on particle motion with sandwich format using procalcitonin as a model system. Ab–DNA conjugates were hybridized to an antifouling PLL-*g*-PEG coating, and the sensor response was measured with PCT concentrations between 10 pM and 10 nM. The sensor showed less nonspecific interactions for the substrate functionalized with PLL-*g*-PEG and Ab–DNA conjugates in comparison to physically adsorbed Abs. The measured dose–response curves demonstrate the biosensing functionality of the Ab–DNA conjugates on the antifouling polymer.

The coupling concepts described in this article are relevant for the development of biosensing platforms that use antibodies as affinity binders. Antibodies show good specificity and stability and are available for many analytes; therefore, the molecules are widely used in the field of biosensing. The coupling strategy of this paper enables oriented coupling to an antifouling substrate. Alternative methods to modify glycans of antibodies to contain an azide group have been described by Kong et al.^37^ and Sadiki et al.^38^ As a next step, the coupling strategy can be tested with different types of antibodies and the sensing performance can be studied with biological fluids. Also, it will be interesting to explore opportunities for multiplexed detection, for instance, by coupling different antibodies to the PLL-*g*-PEG substrate using distinct DNA binders. Furthermore, biofunctionalization could be further studied using advanced microscopic techniques. We believe that the presented oriented coupling methodology of glycan-remodeled Ab–DNA conjugates on antifouling PLL-*g*-PEG-coated substrates will become a flexible route for the design of novel antibody-based biosensors.

## Materials and Methods

### Materials

All chemicals were purchased from Sigma-Aldrich (Netherlands) unless stated otherwise. The enzymes endoglycosidase SH and galactosyltransferase GalT(Y289L) were provided by Synaffix (Netherlands), as well as manganese(II) chloride and UDP-GalNac6N_3_. Anti-PCT antibodies 13B9 and 17A3 were provided by HyTest (Finland). Reagents for gel electrophoresis (tris/glycine/SDS running buffer, Laemmli loading buffer, Coomassie brilliant blue R-250, and precision plus protein standard) were purchased from Bio-Rad (Netherlands). EZlink NHS-PEG_4_-Biotin labels, Novex wedgewell 4–20% tris glycine gels, and Dynabeads MyOne Streptavidin C1 particles were obtained from Fisher Scientific (Netherlands). PLL-*g*-PEG was purchased from SuSoS (Switzerland), and azide-functionalized PLL-*g*-PEG was obtained from Nanosoft Biotechnology LLC. Biotinylated mPEG (1 kDA) was purchased from Nanocs. The ssDNA oligonucleotides were purchased from Integrated DNA Technologies (IDT, Belgium). All reagents were used as received.

### Antibody Modification

Biotinylated Abs were produced using the protocol provided with the EZLink-NHS-PEG_4_-biotin kit. The buffer of the Abs was exchanged to reaction buffer using Amicon Ultra 0.5 mL Ultracel 50K spin filters. Then, a 20-fold molar excess of NHS-PEG_4_-biotin (20 mM) was added to the Abs, and biotinylation was done for 30 min at room remperature (RT). Excess NHS-PEG_4_-biotin was removed using Amicon Ultra 0.5 mL Ultracel 50K spin filters. The biotinylated Abs were aliquoted and stored at −20 °C until further use.

Abs for oriented coupling were modified using a similar procedure as described by Wijdeven et al.^30^ Abs (15 mg/mL) were buffer-exchanged to tris-buffered saline (TBS) and incubated overnight at 30 °C with endo-SH (0.15 mg/mL), whereafter GalT(Y289L) (0.75 mg/mL), UDP-6-N3-GalNac (2.5 mM), and MnCl_2_ (10 mM) were added and incubation continued at 30 °C for 9 h. The azido-Abs were purified using Amicon Ultra 0.5 mL Ultracel 10K spin filters. Four equivalents of DBCO-ssDNA (5′ CGA TTC GAG AAC GTG ACT GCT TTT T 3′-DBCO) were added to the Abs and allowed to react overnight at 30 °C. Unbound DNA was removed using Amicon Ultra 0.5 mL Ultracel 50K spin filters. Ab–DNA conjugates were stored at −20 °C until further use.

### Electrophoresis

Abs (0.5 mg/mL) were reduced for 30 min at 56 °C using an equivalent dithiothreitol (DTT) (30 mg/mL), whereafter they were incubated with 2 equiv of Laemmli loading buffer for 10 min at 95 °C. Nonreduced Abs were incubated with 1 equiv of phosphate-buffered saline (PBS) buffer and 2 equiv of Laemmli loading buffer for 10 min at 95 °C. All Abs were loaded on the protein gel, and the gel was allowed to run for 1.5 h at 150 V. After electrophoresis, the gel was incubated for 15 min with Coomassie brilliant blue R-250, followed by overnight washing in ultrapure water. The gels were imaged using a Gel Doc EZ Imager (Bio-Rad) and processed using Image Lab 6.0.1. (Bio-Rad).

### Mass Spectrometry

Abs (1.5 mg/mL) were reduced for 30 min at 56 °C using a half equivalent DTT (30 mg/mL), after which they were diluted to 0.1 ± 0.3 mg/mL using 0.1% formic acid in ultrapure water. All samples were analyzed using a Waters Aquity UPLC I-class Plus liquid chromatograph attached to a Waters Xevo G2-XS QToF mass spectrometer (Milford, MA). For each sample, 0.04–0.1 μg of protein on a column was analyzed. Separation was performed using a Waters BioResolve RP mAb polyphenyl column (450 Å, 2.7 μm 2.1 × 100 mm^2^), thermostated at 80 °C, operated at a flow rate of 0.4 mL/min with mobile phases (A) 0.1 (v/v)% formic acid in ultrapure water and (B) 0.1 (v/v)% formic acid in acetonitrile. A gradient was set to start at 15% B, increase to 55% B in 10 min, kept constant for 1 min, increase to 95% B in 30 s, kept constant for 1 min, and decrease to 15% B in 10 s, which was retained to reach a final gradient time of 15 min. Ionization and mass analysis was achieved using electrospray ionization in positive ion mode (ESI+) operated with capillary voltage at 0.8 kV, sample cone voltage at 70 V, source offset at 80 V, source temperature at 125 °C, desolvation temperature at 600 °C, cone gas flow at 10 L/h, and desolvation gas flow at 600 L/h. The devices were controlled by MassLynx Software (version 4.2, Waters). Prior to each batch, the system was calibrated using cesium iodide (CsI) over the measurement range of 350–5000 *m*/*z*. During each measurement, the system was calibrated using LeuEnk lock mass (556.27 *m*/*z*) with 30 s intervals. Exact masses and peak intensities were obtained by deconvolution using the MaxEnt 1 tool in MassLynx (v4.2) using the five most abundant charge states.

### Antibody Orientation

The orientation of Ab–DNA conjugates and biotinylated antibodies was assessed using the method described by Tholen et al.^34^ Carboxyl-functionalized silica particles (Bangs Laboratories, 1 μm mean diameter) were functionalized with streptavidin using a protocol previously described by Lubken et al.^39^ 10 mg/mL particles were activated using EDC at a final concentration of 4.3 mM and NHS at a final concentration of 10 mM in 2-(*N*-morpholino)ethanesulfonic acid (MES) buffer (0.1 M MES at pH 5.0) for 30 min at RT. Following incubation, the particles were washed centrifugally at 6000*g* for 5 min and resuspended in MES buffer. Streptavidin, dissolved in ultrapure water, was added to the particles at a final concentration of 66.7 μM. The NHS–streptavidin conjugation was performed overnight at RT. The streptavidin-functionalized silica particles were washed twice using TBS-Tween buffer (25 mM Tris–HCl, 0.15 mM NaCl, 0.05 vol % Tween-20) and twice using 0.1 wt % bovine serum albumin (BSA) in PBS-Tween buffer (130 mM NaCl, 7 mM Na_2_HPO_4_, 3 mM NaH_2_PO_4_, and 0.05 vol % Tween-20, at pH 7.4).

Nonoriented coupling of Abs to the particles was achieved by incubating streptavidin-functionalized silica particles (5 mg/mL) with 2.5 vol equiv of biotinylated Abs (200 nM) overnight at 4 °C. Oriented coupling of Abs to the particles was achieved by incubating streptavidin-functionalized silica particles (5 mg/mL) with 5 vol equiv of biotin-DNA (5′ CGA TTC GAG AAC GTG ACT GCT TTT T 3′-DBCO, 10 μM) for 1 h on a rotary fin. The particles were washed twice using PBS and redissolved in Ab–DNA conjugates in PBS (200 nM) to a particle concentration of 5 mg/mL. The particles were incubated overnight on a rotary fin at 4 °C. Blocking of both functionalized particles was achieved by adding an excess of biotin-mPEG (±20 equiv, 100 μM) to the particles. The particles were incubated at RT for 10 min. The functionalized particles were centrifugally washed twice using PBS. Following the last washing step, the particles were resuspended in 1 wt % BSA in PBS to a particle concentration of 0.1 mg/mL and incubated on a rotary fin for 1 h. After incubation, the particles were centrifugally washed once using PBS. The particles were redissolved in PBS to a particle concentration of 0.1 mg/mL, and 3 vol equiv of particles were incubated with 1 vol equiv D1-DNA (3′-ATC TAC ATA TT/5AmMC6/-5′)-functionalized protein G (10 μg/mL) and 1 vol equiv DPS3-DNA (3′-/3AmMO/TTA GGA GGG-5′)-functionalized protein M (15 μg/mL). Subsequently, the particles were incubated for 45 min on a rotary fin, followed by centrifugal washing. The particles were redissolved in PBS to a final particle concentration of ∼1 mg/mL. The particles were added to an Ibidi μ-slide VI 0.5 glass bottom slide and allowed to sediment for 15 min. The flow cells were washed using buffer B+ (5 mM Tris–HCl, 10 mM MgCl_2_, 1 mM ethylenediaminetetraacetic acid (EDTA), 0.05% v/v Tween 20, pH 8) and imagers IPS3-Cy3B (5′-/Cy3B/TCC TCC C-3′,1 nM) and I1-ATTO647N (5′-CTA GAT GTA T/3ATTO647NN/-3′, 1.6 nM) were added. DNA-PAINT images were obtained using a Bruker Vutara VXL. Fluorescence was recorded in biplane mode using a 60*x*/1.3-numerical aperture objective, passed through a beam splitter, and collected using an Orca Flash 4.0 V3 sCMOS camera. Images were acquired in multiple fields of view of 50 × 50 μm^2^, sequentially imaging using a 555 nm laser with 15 ms exposure time and a 638 nm laser with 10 ms exposure time. Images were recorded at 14 *Z*-positions for 1200 frames at each *Z*-position for both probes subsequently.

## Biosensing by Particle Motion

### Substrate Functionalization

Physical adsorption of Abs to glass slides (25 × 75 mm^2^ #5, Menzel-Gläser) was achieved by first sonicating the slides for 30 min in isopropanol, followed by 10 min of sonication in ultrapure water. The slides were dried using a nitrogen stream, a custom-made flow cell sticker (Grace Biolabs) with an approximate volume of 20 μL was attached to the slides, and anti-PCT 13B9 (100 nM) was introduced into the flow chamber. After 1 h of incubation, the flow cell was washed using PBS, and 1% m/v BSA in PBS was injected in the flow chamber for blocking, which was replaced after 1 h with assay buffer (0.1 wt % BSA in PBS).

For oriented coupling of Abs, a PLL-*g*-PEG coating was adsorbed on a polystyrene slide (25 × 75 × 1.2 mm^3^, Goodfellow GmbH). The slide was first treated with oxygen plasma for 1 min, whereafter the custom-made flow cell sticker was attached immediately, directly followed by injection of a PLL-*g*-PEG with PLL-*g*-PEG/N_3_ (0.45/0.05 mg/mL) mixture. After 3 h of incubation, unbound PLL-*g*-PEG with PLL-*g*-PEG/N_3_ was removed from the flow chamber by removing the fluid in the chamber, and a mixture of DBCO-ssDNA (5′ CGA TTC GAG AAC GTG ACT GCT TTT T 3′-DBCO, 10 μM) and ssDNA (5′AAA AAG CAG TCA CGT TCT CGA ATC GCG TGA GTC CGT CGA CGG ACT 3′) in high salt buffer (0.5 M NaCl in PBS) was added and incubated for at least 3 days. After incubation, unbound DNA was washed away using high salt buffer, and a third ssDNA (5′ AAA AAG CAG TCA CGT TCT CGA ATC GAG TCC GTC GAC GGA CTC ACG 3′,10 μM) was added to the fluid chamber and incubated for 30 min, after which the flow cell was washed using high-salt buffer and an Ab–DNA conjugate (0.17 μM) was injected and incubated for one and a half hour. Unbound conjugates were washed away, and assay buffer (0.1 wt % BSA in PBS) was injected in the flow cell.

### Particle Functionalization

Particles were functionalized using biotinylated anti-PCT 27A3. An equal volume equivalent of Dynabeads (10 mg/mL) and antibodies (100 nM) was incubated for 45 min on a rotary fin. An excess of biotin-mPEG (±20 vol equiv, 100 μM) was added to the particles, and incubation was proceeded for 10 min. Particles were washed with PBS using a magnetic rack, whereafter the particles were redissolved in 1 wt % BSA in PBS to a particle concentration of 0.1 mg/mL and incubated on a rotary fin for 1 h. After blocking, the particles were sonicated (Hielscher, Ultrasound Technology) using 10 pulses at an amplitude of 70 and duty cycle of 0.4 s. The particles were further diluted in 0.1 wt % BSA in PBS to 5 μg/mL.

### Biosensing by Particle Motion Experiment

For the BPM measurement, particles were injected in the flow chamber (either with physically adsorbed or oriented coupled Abs on the substrate) and were allowed to sediment for 15 min. Particle motion was measured in blocks of 5 min, with a frame rate of 60 Hz, using a Leica DMI5000M microscope (dark-field microscopy, Leica Microsystems, Germany) equipped with a high-speed FLIR camera (Sony Grasshopper 3; GS3-U3-23S6M-C) with a field of view of 1920 × 1200 pixels (1129 × 706 μm^2^). Particle tracking software developed in house was used to analyze the particle trajectories and to determine the diffusivity of the particles using MATLAB (2021a). Particles with a diffusion coefficient >0.15 μm^2^/s were considered unbound, and particles with a diffusion coefficient of <0.15 μm^2^/s were considered bound. Data were visualized using Origin (Pro), version 2022, OriginLab Corporation, Northampton, MA.

## Supporting Information Available

Modification of trastuzumab, modification of anti-PCT antibody 13B9, orientation of coupled antibodies, and procalcitonin BPM sensor (PDF)

## Abbreviations

Ab: antibody
Abs: antibodies
BPM: biosensing by particle motion
DNA-PAINT: DNA point accumulation for imaging in nanoscale topography
HC: heavy chain
LC: light chain
LC-MS: liquid chromatography/mass spectrometry
MSD: mean square displacement
PLL-*g*-PEG: poly(l)-lysine-*grafted*-poly(ethylene glycol)
SDS-PAGE: sodium dodecyl sulfate polyacrylamide gel electrophoresis
SD: standard deviation
SEM: standard error of the mean
PCT: procalcitonin
