# Correction to “Oriented Antibody Coupling to an Antifouling Polymer Using Glycan Remodeling for Biosensing by Particle Motion”

**DOI:** 10.1021/acs.bioconjchem.4c00469

**Published:** 2024-10-28

**Authors:** Maud D. M. E. Linssen, Yu-Ting Lin, Sebastian A. H. van den Wildenberg, Marrit M. E. Tholen, Arthur M. de Jong, Menno W. J. Prins

In the original manuscript, an error was present in the Materials section, in the sentence “Anti-PCT antibodies 13B9 and 17A3 were provided by HyTest (Finland).” Here, 17A3 is not correct. The correct antibody is 27A3.

Furthermore, in the Acknowledgments section, essential contributions were inadvertently omitted. The Acknowledgments are as shown below.

