# Supplementary material for: Oriented Antibody Coupling to an Antifouling Polymer Using Glycan Remodeling for Biosensing by Particle Motion
Source: Bioconjug Chem. 2024 Jul 1;35(7):996–1006. doi: 10.1021/acs.bioconjchem.4c00196 (PMC11261616; doi:10.1021/acs.bioconjchem.4c00196)
Supplement: Supplementary file 1 — bc4c00196_si_001.pdf [file bc4c00196_si_001.pdf]

Supplementary Materials for

Oriented Antibody Coupling to an Anti-Fouling Polymer using Glycan Remodeling for Biosensing by Particle Motion

Maud D. M. E. Linssen[1,2], Yu-Ting Lin[3,4], Sebastian A. H. van den Wildenberg[1,2], Marrit Tholen[1,2], Arthur M. de Jong[2,3], Menno W. J. Prins[1,2,3,4]*

[1]Department of Biomedical Engineering, Eindhoven University of Technology, the Netherlands.
[2]Institute for Complex Molecular Systems (ICMS), Eindhoven University of Technology, the Netherlands.
[3]Department of Applied Physics, Eindhoven University of Technology, the Netherlands.
[4]Helia Biomonitoring, Eindhoven, the Netherlands.

Contents

1. List of Supplementary Figures and Tables 2
2. Modification of trastuzumab 3
3. Modification of anti-PCT Antibody 13B9 6
4. Antibody coupling orientation 7
5. Procalcitonin assay in BPM 9

1. List of Supplementary Figures and Tables

Supplementary Figures

Figure S1. Trastuzumab glycan remodeling at different stages; Mass spectra. 4
Figure S2. Trastuzumab glycan remodeling at different stages; Gel electrophoresis 5
Figure S3. Ab-DNA conjugation yield for different equivalents DBCO-ssDNA 5
Figure S4. XZ views of particles with two biofunctionalization methods 8
Figure S5. Diffusivity histograms for two substrate functionalizations 9

Supplementary Tables

Table S1. Modification yield of anti-PCT antibody 13B9..6
Table S2. Conjugation yield of anti-PCT antibody 13B9...6

2. Modification of trastuzumab

The protocol for glycan remodeling was provided by Synaffix. The protocol was executed as received, without optimizations. Initially, trastuzumab was modified and analyzed to master the protocol, optimize analytical protocols, and optimize DBCO-ssDNA ratio for click-coupling. Trastuzumab is a human IgG1 kappa antibody that is used in the treatment of breast cancer due to its interfering properties with HER2 [1]. The antibody was used because of its easy availability and similarity in antibody type to the anti-procalcitonin-antibody used in the paper, which is also an IgG1 antibody. Both trastuzumab and anti-procalcitonin-antibody 13B9 contain glycosylation sites only in the Fc domain.

LC-MS analyses of the different modification steps of trastuzumab (fig. S1) show similar results as the modification of anti-PCT 13B9 (fig. 2). The mass spectrum of unmodified reduced Abs showed the presence of glycans at the heavy chains (HC), with masses ranging from 50.550 Da to 50.940 Da (fig. S1A). After glycan removal using endoglycosidase SH, the MS spectrum shows a reduction in mass of the HC, showing peaks corresponding to the presence of only one N-acetylglucosamine (49.352 Da) or one N-acetylglucosamine with a fucose (49.499 Da) (fig. S1B). Following attachment of GalNac6N_3, the mass of the main peaks shifted with 203 Da to 49.554 Da and 49.701 Da for the glycans with and without fucose present respectively, corresponding to the mass addition of GalNac6N_3 of which the azides are reduced by DTT (fig. S1C). The MS spectrum also shows small peaks at the masses of HC's without GalNac6N_3 present, indicating that GalNac6N_3 was not attached to all glycosylation sites. To determine the yield of glycan attachment, the peak area's with and without GalNac6N_3 were compared. Glycan remodeling yield was calculated following equation S1:

(S1) $$Modification\ yield = \frac{I_{GalNac6N3}}{I_0 + I_{GalNac6N_3}} \cdot 100\%\ ,$$

in which $I_{GalNac6N3}$ is the peak area of the HC with GalNac6N_3 attached and I_0 is the peak area of the HC without GalNac6N_3. The glycan remodeling yield is approximately 81%.

Gel-electrophoresis was performed of reduced Abs to determine the Ab-DNA conjugation yield of Trastuzumab (Fig. S2). The gel shows the Abs at the different stages of the modification together with controls containing the enzymes without Abs. All controls (lanes 3, 5, and 7) show no signal, indicating no interference of the enzymes or DNA in the gels. In comparison to the unmodified Ab (lane 1), the Abs shows a small reduction in size after glycan removal (lane 2), and no change in size after GalNac6N_3 attachment (lane 4), as the change in mass is too small to be observable in gel electrophoresis. After attachment of DBCO-ssDNA, a band appears at ~73 kDa, indicating successful attachment of DNA to a fraction of the Abs (lane 8). Non-specific attachment of DBCO-ssDNA to the Ab does not occur, as this band at ~73 kDa is not observed when intact Abs are incubated with DBCO-ssDNA (lane 6).

Next, the Ab-DNA conjugation yield was optimized by incubating antibodies with various equivalents of DBCO-ssDNA and analyzing the conjugates using SDS-PAGE (fig S3). DBCO-ssDNA to Ab equivalents ranging from 2 to 10 were used. The Ab-DNA conjugation yield was determined using the relative intensity of the two bands of unconjugated Abs and Ab-DNA conjugate at ~50 kDa (I_{Ab}) and ~73 kDa ($I_{Ab\text{-}DNA\ conjugate}$) respectively following equation S2:

(S2) $$Conjugation\ yield = \frac{I_{Ab\ -\ DNA\ conjugate}}{I_{Ab} + I_{Ab\ -\ DNA\ conjugate}} \cdot 100\%.$$

A maximum conjugation yield between 60% and 64% was reached at 4 equivalents of DBCO-ssDNA, and did not increase with additional DBCO-ssDNA.

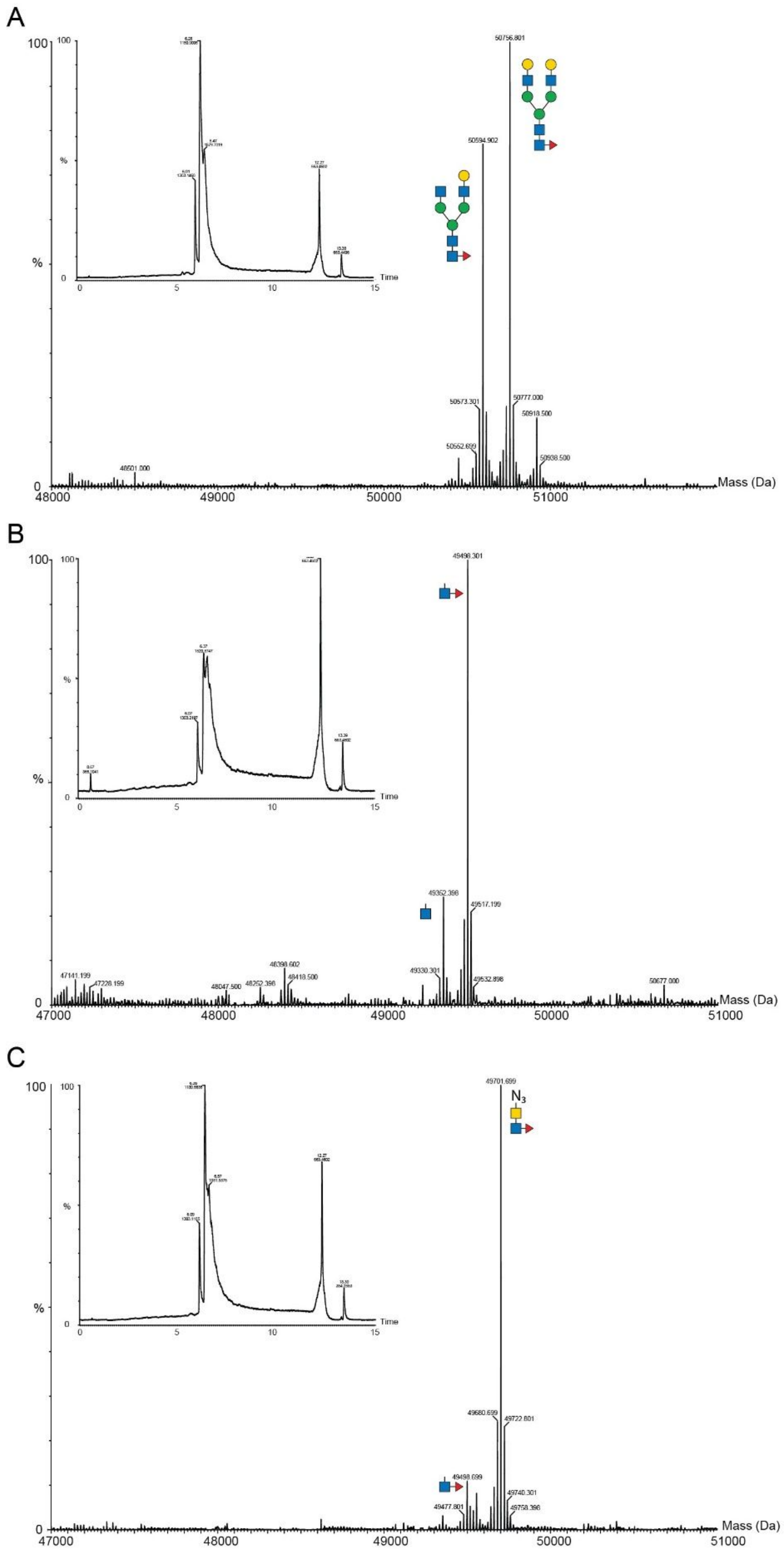

Figure S1. ***Trastuzumab glycan remodeling at different stages; Mass spectra.*** *Deconvoluted mass spectra of heavy chain (HC) fragments of Abs at different stages of the glycan remodeling. Glycan structures are indicated next to the most prominent peaks. The inserts show the chromatograms of the corresponding mass spectra. A) HC of reduced trastuzumab with intact glycosylation sites. B) HC of reduced trastuzumab after glycosylation. C) HC of reduced trastuzumab with GalNac6N_3 coupled to the glycosylation site. The peak intensity at 49.701 Da is $4.6 \cdot 10^6$ and at 49.497 Da is $1.1 \cdot 10^6$. The mass spectra are taken from the components with an LC retention time between 6 and 8 minutes.*

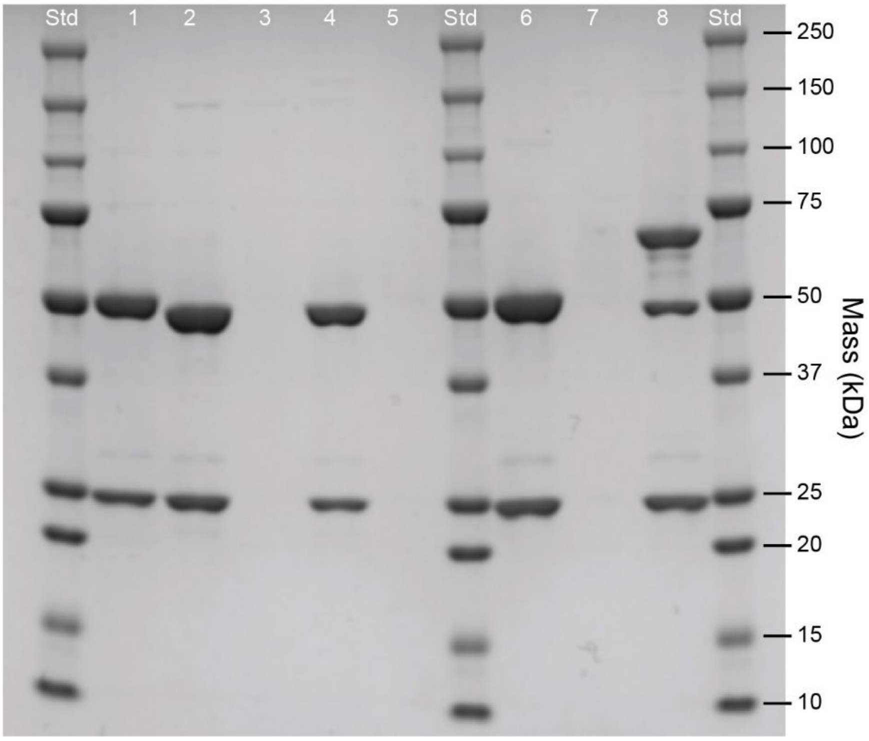

Figure S2. ***Trastuzumab glycan remodeling at different stages; Gel electrophoresis***. *Bands at ~50 kDa indicate the HC, at ~26 kDa indicate the LC, and at ~73 kDa indicate the HC-DNA conjugate. 1) Trastuzumab; 2) Trastuzumab after deglycosylation; 3) deglycosylation blank; 4) Trastuzumab with GalNac6N$_3$ attached; 5) Glycan attachment blank; 6) Unmodified Trastuzumab incubated with DBCO-ssDNA; 7) DBCO-ssDNA conjugation blank; 8) Trastuzumab Ab-DNA conjugate with relative intensities of 0.43 and 0.8 of the bands at ~50 and ~73 kDa respectively.*

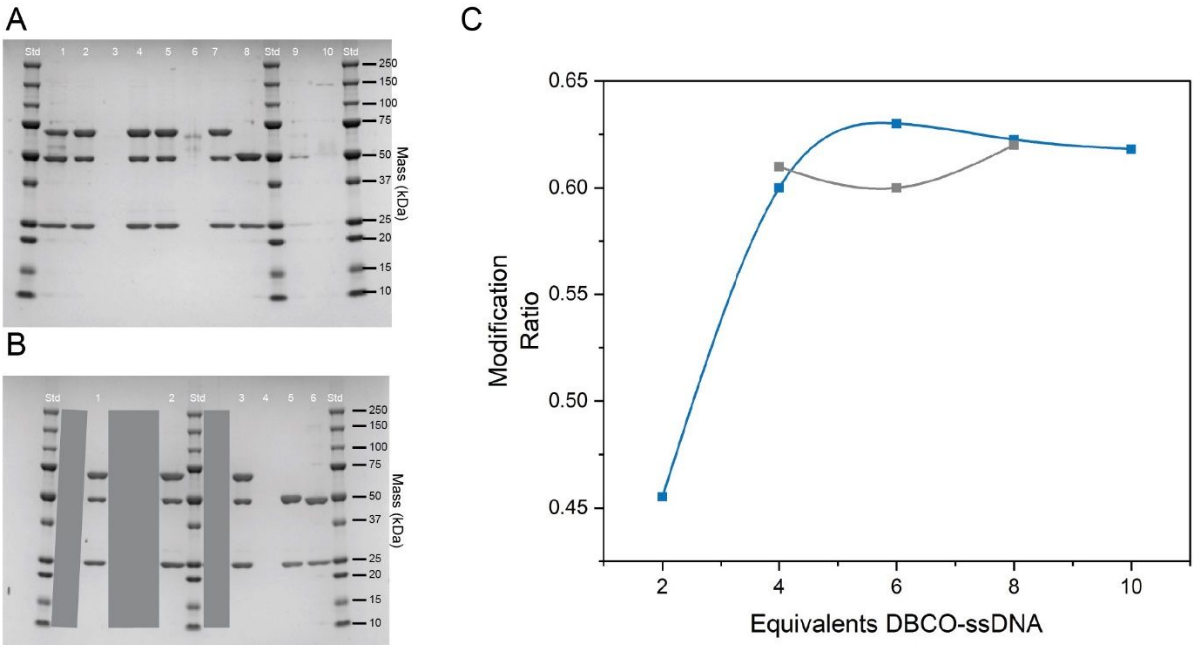

Figure S3: ***Ab-DNA conjugation yield for different equivalents DBCO-ssDNA***. *A) and B) gels of reduced Ab for which glycan remodeled Trastuzumab was incubated with two (A1), four (A2, B1), six (A4, B2), eight (A5, B3), and ten (A7) equivalents of DBCO-ssDNA. Lanes A3, A6 and B4 correspond to blanks and lanes A8, B5 and B6 correspond to unmodified Trastuzumab. Bands at ~50 kDa indicate the HC, at ~26 kDa indicate the LC, and at ~73 kDa indicate the HC-DNA conjugate. C) conjugation yields of the Ab-DNA conjugates in gel A (blue line) and gel B (grey line), calculated using equation S2.*

3. Modification of anti-PCT Antibody 13B9

Modification efficiencies of the anti-procalcitonin antibody 13B9 are discussed in the results section. Figure 2A3 shows the mass spectrum of Abs to which GalNac6N_3 is attached. This sample was measured in quintuplicate, for which the peak areas at 49.648 and 49.444 Da, shown in table S1, were measured and used to determine the modification yield using equation S1. The modification yield (+SEM) of anti-PCT antibody 13B9 is 80.6 ± 0.5%.

The conjugation yield of anti-PCT antibody 13B9 to DBCO-ssDNA was analyzed using gel electrophoresis, for which results are shown in figure 2B. Gel electrophoresis was repeated to accurately determine the conjugation yield, for which the results are shown in table S1. The conjugation yield (+SEM, calculated using equation S2) of anti-PCT-antibody 13B9 to DBCO-ssDNA is 33 ± 2%.

*Table S1. **Modification yield of anti-PCT antibody 13B9**. Relative peak area of the masses 49.648 and 49.444 measured in the mass spectra of reduced, remodeled anti-PCT antibody 13B9. The peaks correspond to the antibody without and with GalNac6N_3, respectively. The modification yield was calculated using equation S1.*

	Peak area at 49.648 Da ($\cdot 10^7$)	Peak area at 49.444 Da ($\cdot 10^7$)	Modification yield (%)
1	8.30	2.24	78.8
2	8.38	1.95	81.1
3	8.28	1.93	81.1
4	8.08	1.94	80.7
5	7.96	1.81	81.5

*Table S2. **Conjugation yield of anti-PCT antibody 13B9.** Relative intensities of the bands detected at ±50 kDa and ±75 kDa, corresponding to HC and HC-DNA conjugate respectively, in various gel electrophoresis experiments used to determine the percentage of HC antibody fragments that are conjugated to DNA. The conjugation yield was calculated using equation S2.*

	Relative intensity 50 kDa	Relative intensity 75 kDa	Conjugation yield (%)
1	50.2	18.6	27
2	41.2	22.6	35
3	37.8	18.5	33
4	48.7	29.2	37

4. Orientation of coupled antibodies

Antibodies coupled to silica particles (1 µm diameter) were imaged in 3D using single-molecule DNA-PAINT microscopy. For each particle the number of localizations in the green and red fluorescence channels were counted. Particles on the edges of the FOV were excluded from analysis because at the edges the inhomogeneous laser intensities resulted in significant differences in the observed number of localizations. The XZ views of particles used to construct fig. 3D are shown in fig. S4. Particles functionalized with biotinylated Abs show 77.8 ± 9.1 % (±SD) green localizations and particles functionalized with Ab-DNA conjugates show 99.5 ± 0.6 % (±SD) green localizations.

The experiments show small particle-to-particle variations in a single measurement. Different experiments resulted in different densities of particles on the substrate. Due to the limited availability of the equipment, only a few experiments could be performed.

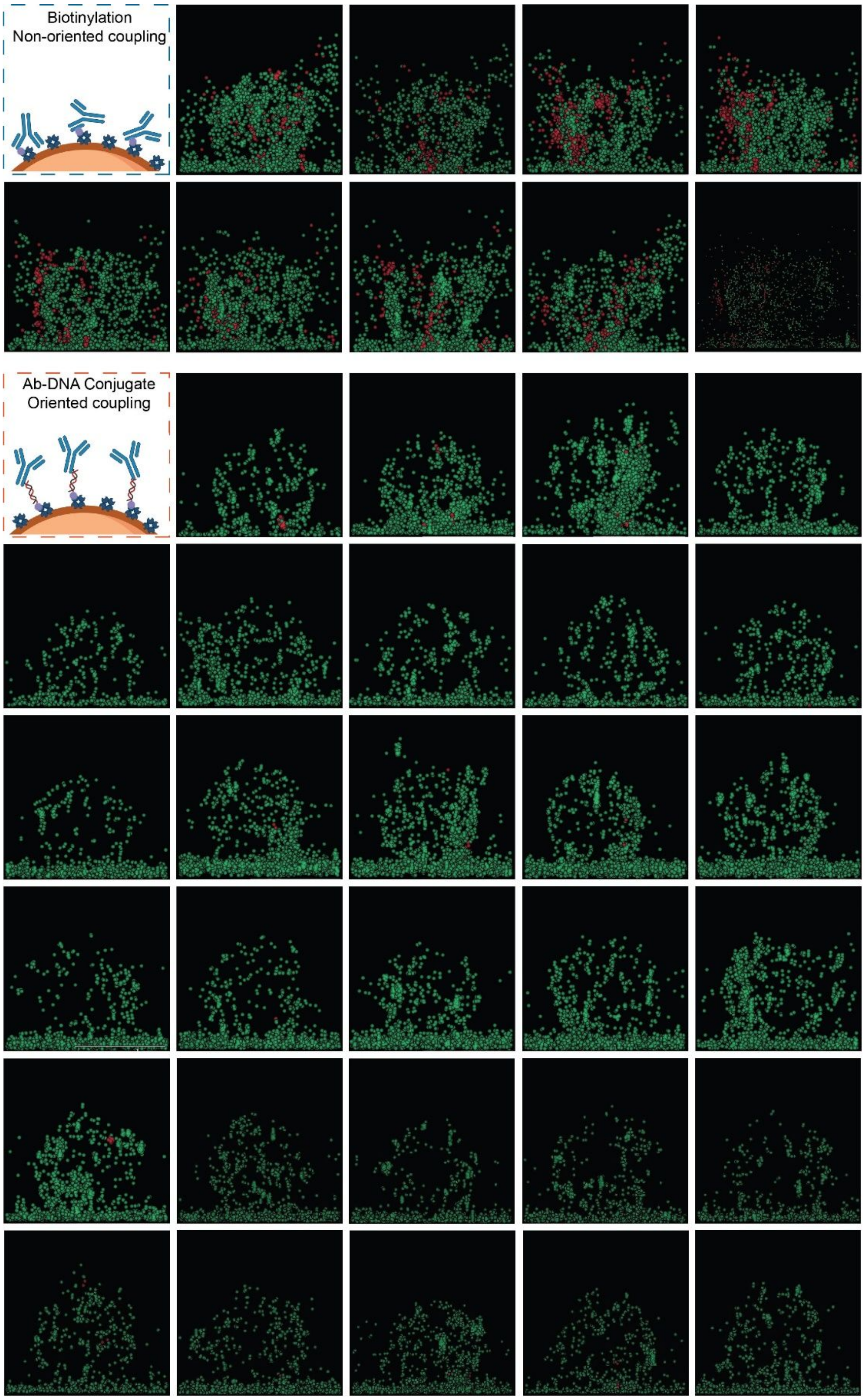

Figure S4. ***XZ views of particles with two biofunctionalization methods****: biotinylated Abs (top) and Ab-DNA conjugates (bottom). Green localizations indicate protein M binding to Fab sites and red localizations indicate protein G binding to Fc sites.*

5. Procalcitonin BPM sensor

BPM using Ab-DNA conjugates functionalized on a PLL-g-PEG coating on a polystyrene substrate was compared to physical adsorption of Abs on a glass surface. Figure S5 shows the diffusivity histograms of both functionalization strategies with serial additions of PCT to the sensor. The data shown in the red panel in Figure S5 is the same as for Figure 4C.

The sensor with physically adsorbed antibodies shows that many particles are already bound without PCT, indicating non-specific interactions. With increasing PCT concentrations, the fraction of bound particles rapidly increases and at 333 pM almost all particles are stuck. There is an abrupt change of freely moving particles at 111 pM to almost all particles stuck at 333 pM, indicating that non-specific binding of PCT might play a role. The sensor functionalized with PLL-g-PEG and Abs conjugates shows a much lower signal in the absence of PCT, indicating that there are less non-specific interactions. Furthermore, the sensor response increases more gradually and over a larger concentration range, which suggests that non-specific interactions are reduced with this coupling methodology.

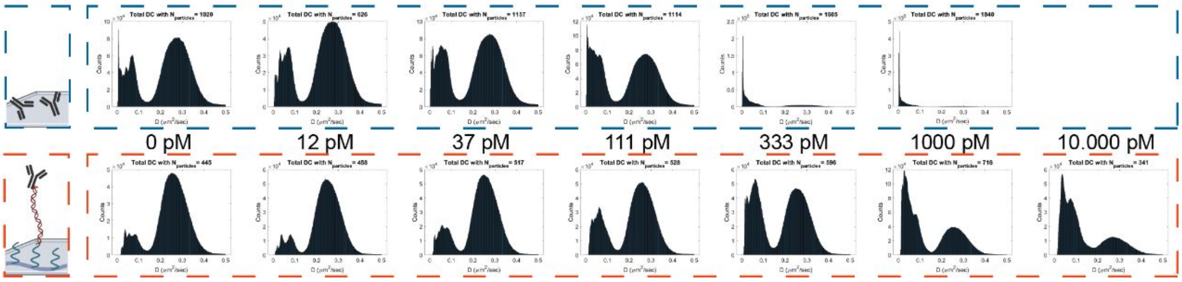

*Figure S5. **Diffusivity histograms for two substrate functionalizations**; physically adsorbed Abs to a glass substrate (top, blue) and Ab-DNA conjugates coupled to PLL-g-PEG on a polystyrene substrate (bottom, red). Diffusivity histograms are shown for increasing PCT concentrations with serial additions of PCT.*
